# Towards the Small and the Beautiful: A Small Dibromotyrosine Derivative from *Pseudoceratina* sp. Sponge Exhibits Potent Apoptotic Effect through Targeting IKK/NFκB Signaling Pathway

**DOI:** 10.3390/md11093168

**Published:** 2013-08-26

**Authors:** Jui-Hsin Su, Yu-Cheng Chen, Mohamed El-Shazly, Ying-Chi Du, Chiang-Wen Su, Chia-Wei Tsao, Li-Lian Liu, Yalan Chou, Wen-Been Chang, Yin-Di Su, Michael Y. Chiang, Yao-Tsung Yeh, Mei-Chin Lu

**Affiliations:** 1 National Museum of Marine Biology & Aquarium, Pingtung 944, Taiwan; E-Mails: x2219@nmmba.gov.tw (J.-H.S.); j520c@hotmail.com (Y.-C.C.); gaway4297@yahoo.com.tw (C.-W.T.); wenbeen@nmmba.gov.tw (W.-B.C.); gobetter04@yahoo.com.tw (Y.-D.S.); 2 Graduate Institute of Marine Biotechnology, National Dong Hwa University, Pingtung 944, Taiwan; E-Mail: mynameisr4@yahoo.com.tw; 3 Graduate Institute of Natural Products, College of Pharmacy, Kaohsiung Medical University, Kaohsiung 807, Taiwan; E-Mails: elshazly444@googlemail.com (M.E.-S.); ycdu0626@gmail.com (Y.-C.D.); 4 Department of Pharmacognosy and Natural Products Chemistry, Faculty of Pharmacy, Ain-Shams University, Organization of African Unity Street, Abassia, Cairo 11566, Egypt; 5 Institute of Marine Biology, National Sun Yat-sen University, Kaohsiung, Taiwan; E-Mails: lilian@mail.nsysu.edu.tw (L.-L.L.); ylchou@gmail.com (Y.C.); 6 Department of Marine Biotechnology and Resources and Asia-Pacific Ocean Research Center, National Sun Yat-sen University, Kaohsiung 804, Taiwan; 7 Department of Chemistry, National Sun Yat-sen University, Kaohsiung 804, Taiwan; E-Mail: michael@faculty.nsysu.edu.tw; 8 Department of Medical Laboratory Sciences and Biotechnology, Fooyin University, Kaohsiung 831, Taiwan; E-Mail: glycosamine@yahoo.com.tw

**Keywords:** apoptosis, dibromotyrosine, mitochondrial dysfunction, oxidative stress, topoisomerase

## Abstract

A dibromotyrosine derivative, (1′*R*,5′*S*,6′*S*)-2-(3′,5′-dibromo-1′,6′-dihydroxy-4′-oxocyclohex-2′-enyl) acetonitrile (DT), was isolated from the sponge *Pseudoceratina* sp., and was found to exhibit a significant cytotoxic activity against leukemia K562 cells. Despite the large number of the isolated bromotyrosine derivatives, studies focusing on their biological mechanism of action are scarce. In the current study we designed a set of experiments to reveal the underlying mechanism of DT cytotoxic activity against K562 cells. First, the results of MTT cytotoxic and the annexin V-FITC/PI apoptotic assays, indicated that the DT cytotoxic activity is mediated through induction of apoptosis. This effect was also supported by caspases-3 and -9 activation as well as PARP cleavage. DT induced generation of reactive oxygen species (ROS) and the disruption of mitochondrial membrane potential (MMP) as indicated by flow cytometric assay. The involvement of ROS generation in the apoptotic activity of DT was further corroborated by the pretreatment of K562 cells with *N*-acetyl-cysteine (NAC), a ROS scavenger, which prevented apoptosis and the disruption of MMP induced by DT. Results of cell-free system assay suggested that DT can act as a topoisomerase II catalytic inhibitor, unlike the clinical anticancer drug, etoposide, which acts as a topoisomerase poison. Additionally, we found that DT treatment can block IKK/NFκB pathway and activate PI3K/Akt pathway. These findings suggest that the cytotoxic effect of DT is associated with mitochondrial dysfunction-dependent apoptosis which is mediated through oxidative stress. Therefore, DT represents an interesting reference point for the development of new cytotoxic agent targeting IKK/NFκB pathway.

## 1. Introduction

Living organisms in oceans are widely regarded as the ancestral tree of all creatures and the cradle of the first signs of uni- and multicellular life. One of the earliest manifestations of multicellular organisms is the development of marine sponges. It took millennia to develop multicellular organisms equipped with necessary tools of survival. Sponges are sessile creatures which can be attacked by different kinds of predators and fouling microorganisms [1]. These harsh conditions led to the development of a highly sophisticated defense system of secondary metabolites. A silver line had to be established in developing such defense system, through synthesizing compounds which are extremely toxic to the predators under high dilution condition, however still tolerable by the sponges. Among these compounds which were discovered in the seventies of the last century, are bromotyrosine derivatives. These derivatives are biosynthesized from common l-tyrosine metabolites such as 3-bromo-l-tyrosine and 3,5-dibromo-l-tyrosine which serve as the basic structural elements for this class of marine alkaloids [2].

In the past four decades, over 300 different bromotyrosine derivatives were isolated from sponges and their biological activity was evaluated [3]. In 1987, a dibromotyrosine derivative was found to act on the hypothalamic centers which control thyroid function and was proposed as a potential candidate targeting certain CNS disorders [4]. Aeroplysinin-1, a dibromotyrosine acetonitrile derivative, was isolated from marine sponges along with an array of closely related analogues. The potent biological activities of these derivatives led to the development of different synthetic protocols for their synthesis [5,6,7]. Reports indicated that aeroplysinin-1 exhibited a myriad of biological activities including antiparasitic [5], antiinflammatory [8], antibiotic [9] and cytotoxic activities [7,10]. Moreover, it was found that this compound can be utilized as a structural template for the synthesis of antiangiogenic agents based on the docking experiments in the ATP binding site of Vascular Endothelial Growth Factor Receptors (VEGFR) [11]. Despite the wide spectrum of biological activities exhibited by bromotyrosine derivatives, only a handful of reports have focused on studying their biological mechanism of action [3]. 

**Figure 1:**
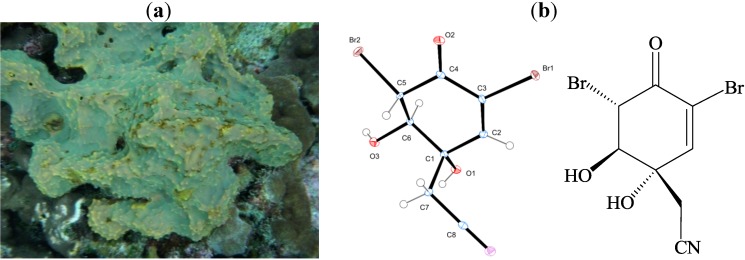
(**a**) Morphology of the Taiwanese Green Island sponge, *Pseudoceratina* sp.; (**b**) Molecular structure of (1′*R*,5′*S*,6′*S*)-2-(3′,5′-dibromo-1′,6′-dihydroxy-4′-oxocyclohex-2′-enyl) acetonitrilebased on X-ray analysis.

Sponges belonging to the order Verongida are considered the richest source of naturally occurring bromotyrosine derivatives. Over 15 classes of these derivatives were isolated including fistularin, psammaplysin, aerothionin, psammaplin, purealidinand bastadin subtypes [12]. Among the widely studied genera of this order is *Pseudoceratina* sp. From this order different bromotyrosine derivatives were isolated including pseudoceramines A–D and spermatinamine [13], aplysamine 6 [14], ceratinadins A–C [15], as well as 11,19-dideoxyfistularin-3, 11-deoxyfistularin-3 and dibromoverongiaquinol [16]. The wide diversity of the separated bromotyrosine derivatives has encouraged us to subject *Pseudoceratina* sp., collected from the Green Island on the east cost of Taiwan, to an intensive chemical investigation which led to the isolation of (1′*R*,5′*S*,6′*S*)-2-(3′,5′-dibromo-1′,6′-dihydroxy-4′-oxocyclohex-2′-enyl) acetonitrile (DT) (Figure 1). This secondary metabolite is an oxidized derivative of aeroplysinin-1, suggesting that DT may possess similar cytotoxic activity. In the current study we sought to evaluate the cytotoxic activity of DT on chronic myeloid leukemia (CML) (K562) as well as to identify its underlying mechanism of action. 

## 2. Results

### 2.1. DT Inhibits Cellular Growth and Induces Apoptosis in K562 Cells

**Figure 2:**
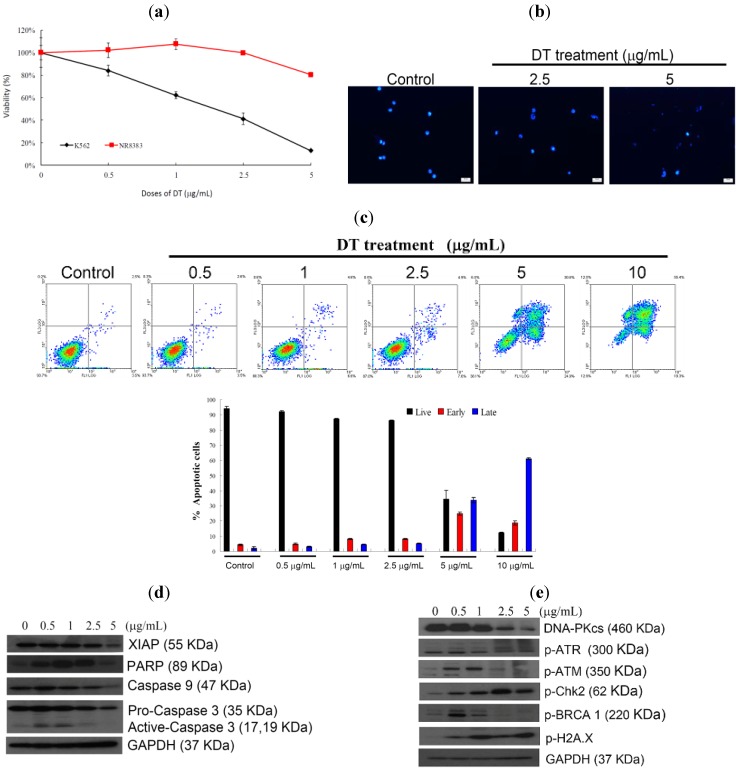
DT inhibits K562 cellular growth and induces apoptosis. K562 cells were treated with different doses of DT and incubated for 24 h, (**a**) Viability of K562 and normal rat macrophage NR8383 cells were determined with different doses of DT; (**b**) Treated cells were stained with DAPI and examined with fluorescence microscope (200×); (**c**) Double staining with annexin V/propidium iodide (PI) was used to determine the induction of apoptosis following the treatment of K562 cells with different concentrations of DT (0, 0.5, 1, 2.5, 5 and 10 µg/mL) for 24 h. The histogram summarizes the apoptotic population in percentage of K562 cells. Results are presented as mean ± SD of three independent experiments; (**d**) Caspase-activated proteins; (**e**) DNA damage-related proteins were examined following the treatment with the indicated doses of DT for 24 h, as shown from the Western blots. GAPDH was the loading control.

The cytotoxic effect of DT was evaluated against several cancer cell lines and we found that DT exhibited potent activity against K562, Hela, MCF-7, and MDA-MB-231 cells, with IC_50_ values of 1.4, 4.8, 1.9, and 5.5 µg/mL, respectively, for 72 h. Leukemia K562 cell line was the most sensitive cell line to the cytotoxic effect of DT and was selected for further investigation. K562 cells and rat alveolar macrophage NR8383 cell line were treated with different doses of DT, for 24 h. DT downregulated viability of K562 cells in a dose dependent manner, but did not suppress growth of NR8383 cells. Even at the highest dose (5 µg/mL), DT treatment resulted in viability of NR8383 cells about 80% (Figure 2a). Nuclear condensation was observed after treating K562 cells with 2.5 and 5 µg/mL of DT (Figure 2b). To understand whether DT cytotoxic effect is mediated through apoptosis, we used annexin V/PI double staining and flow cytometric analysis. We found that increasing the concentration of DT (2.5, 5 and 10 µg/mL) led to a significant increase in the apoptotic population (13, 61.9 and 87.1%, respectively) after 24 h (Figure 2c). Treatment with 0.5 and 1 µg/mL of DT resulted in a significant increase in the level of caspase 3 activation and PARP cleavage, but at high concentration (5 µg/mL) it led to a decrease in caspase 3 activation, PARP cleavage and XIAP expression (Figure 2d). Furthermore, the phosphorylation of H2AX, a biomarker of DNA damage, was observed with the DT treatment. Moreover, an increase in ATM and BRCA phosphorylation was observed with the use of low doses (0.5 and 1 µg/mL) of DT, however their phosphorylation decreased at high doses (2.5 and 5 µg/mL). Also ATR phosphorylation was observed with the treatment of increasing concentrations of DT (Figure 2e). Upon DNA damage, cells activate a complex DNA-damage-response (DDR) signaling network to arrest the cell cycle and repair DNA, but if the extent of the damage is beyond the capacity of the repair system, apoptosis is induced [17]. Our results indicated that apoptosis and DNA damage induced by DT treatment were mediated through caspase activation and Chk2 phosphorylation, respectively.

### 2.2. DT Inhibits Topoisomerase II Catalytic Cycle

**Figure 3:**
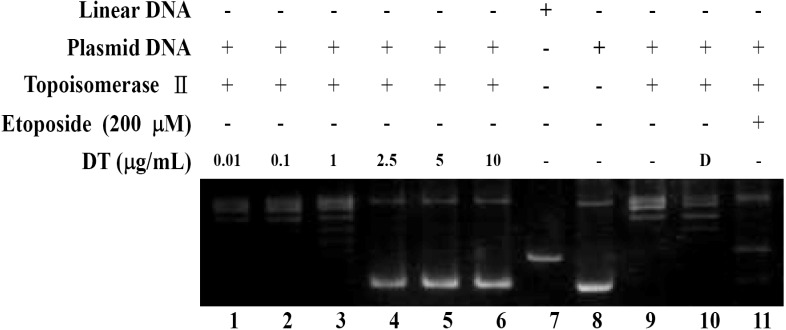
Effect of DT on topo IIα mediated supercoiled pHOT1 plasmid DNA relaxation, Lanes 1–6: DT (0.01, 0.1, 1, 2.5, 5, and 10 µg); Lane 7: Linear DNA; Lane 8: negative control plasmid DNA; Lane 9: plasmid DNA + topoisomerase IIα (induction of DNA relaxation); Lane 10: plasmid DNA + topoisomerase IIα + solvent control (induction of DNA relaxation); Lane 11: positive control, etoposide (20 mM), as topo II poison (induction of linear DNA).

Phosphorylation of H2AX (γH2AX) is the hallmark of DNA damage which was observed in K562 cells upon DT treatment (Figure 2e). We tried to determine whether the DNA damage induced by DT is associated with the interruption of topo II activity. For this purpose, a cell-free DNA cleavage assay using an enzyme-mediated negatively supercoiled pHOT1 plasmid DNA, was applied. As shown in (Figure 3), DT at low doses (0.01, 0.1, and 1 µg) induced DNA relaxation in the presence of topo IIα (Lanes 1–3); but at high doses (2.5, 5, and 10 µg) it inhibited DNA relaxation in the presence of topo IIα (Lanes 4–6). Lane 7 is showing a linear DNA strand which was also observed upon treating the supercoiled pHOT1 plasmid DNA with etoposide, a standard topo II poison (Lane 11) [18]. These results suggested that one of the DT targets, as a DNA damaging agent, is to interfere with various steps of topoisomerase IIα catalytic cycle, which plays a critical role in DNA replication, transcription and chromosomal segregation [19]. 

### 2.3. DT-Induced Apoptosis Is Mediated through Reactive Oxygen Species (ROS) Generation in K562 Cells

**Figure 4:**
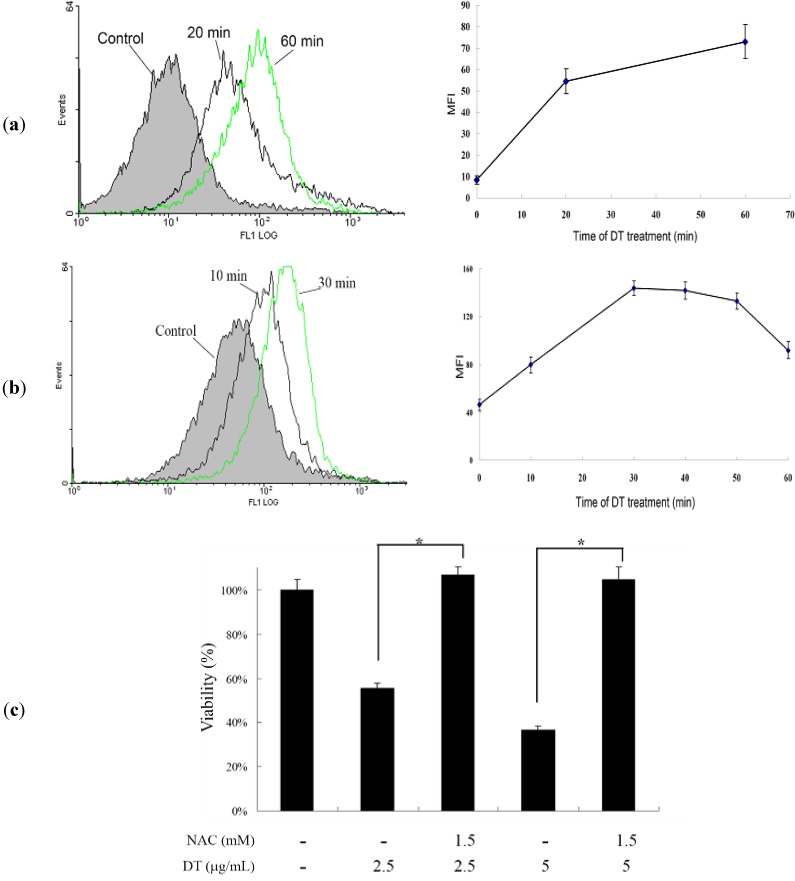
DT-induced K562 cellular apoptosis involves ROS production, we evaluated the effect of DT treatment on the ROS generation in K562 cells; (**a**) Cells were treated with 2.5 µg/mL of DT for the indicated times; (**b**) Cells were treated with 5.0 µg/mL of DT for the indicated times. Quantitative results of the change in the ROS level showed a gradual increase in the ROS production upon DT treatment when compared with the control group; (**c**) Effect of ROS generation on DT-induced cell death in K562 cells. Following pretreatment of 1.5 mM NAC for 1.5 h, cells were treated with 2.5 and 5 µg/mL of DT, respectively, and the cellular viability was measured with trypan blue dye exclusion assay. Results are presented as mean ± SD of three independent experiments (* *P* < 0.001).

Mounting experimental evidence has continued to lend credence to the fact that ROS plays a critical role in the cellular homeostasis and a delicate balance should be established to avoid overproduction of ROS which may induce apoptosis and cell death [20]. Hence, we further examined whether ROS production was involved in the DT-induced apoptosis. Production of ROS was examined utilizing the carboxy derivative of fluorescein, carboxy-H_2_DCFDA. As shown in Figure 4a, treatment with DT at 2.5 µg/mL for 20 and 60 min, resulted in 6.53- and 8.73-fold increase in ROS levels, respectively, and the high production of ROS persisted for the next 18 hours as compared to the mean fluorescence index (MFI) of the control. However, the use of DT (5 µg/mL) for 10, 30, 40, 50 and 60 min resulted in 1.73-, 3.11-, 3.07-, 2.88- and 1.99-folds increase of the fluorescence intensity, respectively as compared to the mean fluorescence index (MFI) of the control (Figure 4b). To clarify whether ROS generation is the major regulator in the DT-induced apoptosis, K562 cells were pretreated with NAC, a ROS scavenger, to suppress the intracellular oxidative stress. Cellular viability was measured by a trypan blue dye exclusion assay following DT treatment. As shown in Figure 4c, NAC pretreatment improved cell viability up to 37% and 58% in response to the use of 2.5 and 5 µg/mL of DT, respectively in comparison to the control group.

### 2.4. DT-Induced Oxidative Stress Disturbs Mitochondrial Membrane Potential (MMP)

**Figure 5:**
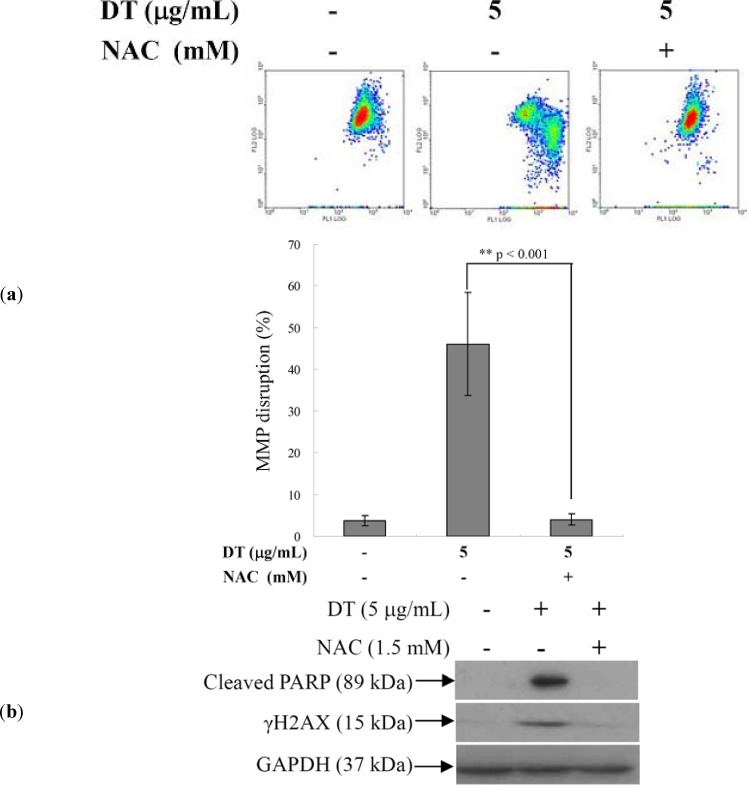
Effect of *N*-acetyl-cysteine (NAC) pretreatment on mitochondrial membrane potential (MMP) disruption- and DNA damage-induced by DT. Following pretreatment with NAC (1.5 mM) for 1.5 h, K562 cells were then treated with 5 µg/mL of DT for additional 24 hours. Cells were collected and analyzed; (**a**) Change of MMP with flow cytometry; (**b**) Expression of DNA damage-related proteins as shown by Western blot, results are presented as mean ± SD of three independent experiments (** *P* <0.001).

The next step after showing that DT induced apoptosis in K562 cells is mediated through ROS overproduction; was to evaluate the effect of ROS overproduction on the mitochondrial membrane potential (MMP) of these cells. Flow cytometric assay with JC-1 cationic dye was used to evaluate this effect. Cells were divided into two groups, one group was treated with NAC (1.5 mM) followed by 5 µg/mL of DT and the other group was treated with DT (5 µg/mL) only. After 24 h, the change of MMP was analyzed in the two groups. DT treatment led to 46.1% disruption of MMP in K562 cells. However, NAC pretreatment maintained the integrity of MMP to the status of the control group (Figure 5a). We further determined the effect of NAC pretreatment on the expression of the DNA damage-signaling proteins. As shown in Figure 5b, NAC pretreatment abrogated PARP cleavage and H2AX phosphorylation.

### 2.5. ROS Generation in DT-Induced Apoptosis is Mediated through the Inhibition of IKK/NFκB and the Activation of PI3K/Akt Pathways

**Figure 6:**
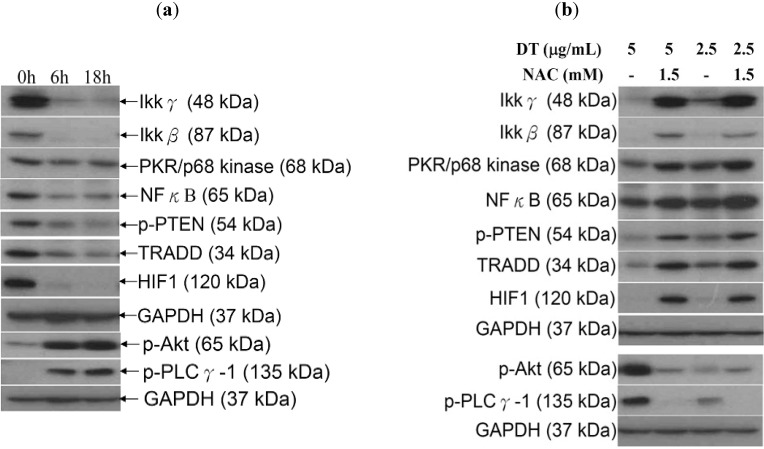
Effect of the oxidative stress caused by DT treatment on IκB kinases (IKK)/NFκB and phosphatidylinositol 3-kinase (PI3K)/Akt pathways; (**a**) Cells were treated with DT (5 µg/mL) for 6 and 18 h, respectively; (**b**) Other group of cells were pretreated with 1.5 mM NAC for 1.5 h, followed by the treatment with 2.5 or 5 µg/mL of DT for 18 additional hours. The expression of IKK/NFκB and PI3K/Akt pathways related proteins was examined in all groups. GAPDH was the loading control.

Recent studies have revealed the interlocking nature of different pathways in controlling several cellular abnormalities such as inflammation and excessive cellular proliferation [21]. It was suggested that IKK/NFκB pathway is a potential therapeutic target in cancer treatment [22,23] and the ROS-mediated apoptosis involves the inhibition of this pathway [24,25]. Additionally, it was found that phosphatidylinositol 3-kinase (PI3K)/Akt (protein kinase B, PKB) signaling pathway is overexpressed in many human malignancies and it plays a critical role in many cellular functions including cellular proliferation and survival, autophagy, metabolism, angiogenesis and motility [26]. Since our results demonstrated that the oxidative stress is the major contributor of DT-induced apoptosis in K562 cells, we further investigated whether these IKK/NFκB and PI3K/Akt pathways are associated with the ROS overproduction. To assess the involvement of IKK/NFκB and PI3K/Akt pathways, Western blot analysis was performed with specific antibodies. This allowed us to examine the effect of DT treatment on the expression of certain proteins related to these pathways. We found that treating K562 cells with DT (5 µg/mL) for 6 and 18 h diminished the expression of IKK/NFκB-related proteins and the phosphorylation of PTEN. On the other hand, an elevation of Akt and PLCγ-1 phosphorylation was observed (Figure 6a). Finally, in line with our expectation, NAC pretreatment of K562 cells abrogated the induction of Akt and PLCγ-1 phosphorylation as well as the inhibition of IKK/NFκB/TRADD, p-PTEN, PKR and HIF 1 expression caused by DT treatment (Figure 6b).

## 3. Discussion

In our preliminary cytotoxic screening assays against different cancer cell lines, DT exhibited a dose- and time-dependent cytotoxic effect against K562 leukemia cell line (IC_50_ of 1.4 µg/mL) (Figure 2a). K562 cells are derived from chronic myeloid leukemia (CML) patients, which are a clonal myeloproliferative disorder with constitutive tyrosine kinase (TK) activity [27,28]. Inhibition of TK has emerged as a promising target for leukemia treatment. The second generation of TK inhibitors, such as nilotinib and dasatinib, has been recently utilized as a potent class of chemotherapeutic agents. However, it was found that these inhibitors possess significant toxicities for 30%–60% of the patients [29], with high possibility of cancer relapse [30]. The significant cytotoxic activity of DT against K562 cells and the lack of efficient treatment for myeloid leukemia (CML) encouraged us to investigate its cytotoxic mechanism of action aiming to develop a safer and more potent anti-myeloid leukemia lead candidate. 

We examined the induction of apoptosis by DT in K562 cells and found that ROS production is a critical factor in regulating DT cytotoxicity. Overproduction of ROS is usually accompanied with negative consequences on cellular function and integrity. It may lead to the disruption of mitochondrial membrane potential (MMP) resulting in the loss of mitochondrial membrane integrity. As shown in Figure 4, Figure 5, pretreatment of K562 cells with NAC, a ROS scavenger, prevented apoptosis and the disruption of MMP induced by DT. A recent report suggested that psammaplysenes A and B, dibromotyrosine-derived metabolites isolated from marine sponges, could act as potent inhibitors of FOXO1a nuclear export [31]. FOXO proteins are both sensors of oxidative stress and effectors of the cellular response [32]. These proteins are important downstream targets of the PI3K/Akt pathway. Activation of PI3K/Akt pathway directly phosphorylates FOXO proteins and inhibits their activity [33]. Our results showed that the DT induced apoptosis can be associated with the inhibition of FOXO, exerted through activation of PI3K/Akt (Figure 6a). 

Reports on new cancer molecular targets indicated that some success has been achieved in the inhibition of PI3K/Akt/mTOR pathway for therapeutic purposes, but unfortunately different feedback loops were found to interrupt chemotherapeutic efficiency of the inhibitors [34]. A recent study has suggested that the loss of PTEN function, a PI3K/Akt related protein, leads to the accumulation of activated Akt, which plays a key role in the PTEN-mediated tumorigenesis via multiple mechanisms including the inhibition of apoptosis [35,36]. Our results were in harmony with previous results showing that the activation of Akt by DT is mediated through the inhibition of PTEN. Despite these preliminary results on the positive role of Akt activation in cancer therapy, its actual role is still controversial. Ahn *et al.* reported that apoptosis induced by shikonin, a secondary metabolite with a naphthoquinone skeleton, required the activation of the Akt/ASK/p38 signaling cascade via ROS generation [37]. On the other hand, chemoresistance accompanying PI3K/Akt activation was found to be regulated by the inhibition of PARP-1 [38] or the promotion of survivin [39]. 

Recently, several preclinical studies have focused on developing novel small molecules as inhibitors of IκB kinases (IKKs), such as Bayer “Compound A” [40], PS-1145 [41], SC-514 [42], and diarylbenzamide [43], aiming to block IKK/NFκB activation [44]. Inhibition of IKKs is emerging as an exciting novel target for cancer therapy [44]. Moreover, hypoxia-Inducible factor-1 (HIF-1) and NFκB were found to act as pivotal regulators of cellular growth and oncogenesis [45]. Interestingly, our results indicated that the inhibition of the ROS generation with NAC pretreatment was accompanied with the recovery of HIF-1 and IKK/NFκB pathway to similar levels of the control group (Figure 6b). Kuphal *et al.* proposed that NFκB regulation in malignant melanoma under normoxic conditions is mediated through ROS. They also demonstrated that the inhibition of NFκB by adenoviral overexpression of NFκB inhibitor (IκB) led to the attenuation of HIF activity [46]. Therefore, we can propose that NFκB-attenuated HIF-1, along with ROS generation, is involved in DT-induced K562 cellular apoptosis.

## 4. Experimental Section

### 4.1. Bioassays Materials

RPMI 1640 medium, fetal calf serum (FCS), trypan blue, penicillin G, and streptomycin were obtained from GibcoBRL (Gaithersburg, MD, USA). 3-(4,5-dimethylthiazol-2-yl)-2,5-diphenyl-tetrazolium bromide (MTT), dimethyl sulfoxide (DMSO), and all other chemicals were purchased from Sigma-Aldrich (St. Louis, MO, USA). Antibodies against caspases-3, -8, and -9,TRADD, PKR, p-PTEN, p-PLCγ-1 as well as p-Akt (Ser^473^) and PARP were purchased from Cell Signaling Technologies (Beverly, MA, USA). Antibodies of Ikkγ, Ikkβ, GAPDH, XIAP, and NFκB (p65) were obtained from Santa Cruz Biotechnology (Santa Cruz, CA, USA). HIF 1 was obtained from Enzo Life Science International, Inc. (Farmingdale, NY, USA). JC-1 cationic dye, and the carboxy derivative of fluorescein (carboxy-H_2_DCFDA) were purchased from Molecular Probes and Invitrogen detection technologies (Carlsbad, CA, USA). Anti-mouse and rabbit IgG peroxidase-conjugated secondary antibody were purchased from Pierce (Rockford, IL, USA). HybondECL transfer membrane and ECL Western blotting detection kits were obtained from Amersham Life Sciences (Amersham,UK).

### 4.2. Preparation of (1′*R*,5′*S*,6′*S*)-2-(3′,5′-dibromo-1′,6′-dihydroxy-4′-oxocyclohex-2′-enyl) Acetonitrile (DT) Stock Solution

The marine sponge *Pseudoceratina* sp. was collected by scuba diving at the Green Island, which is located on the east coast of Taiwan, in March 2011, at a depth of 10 m, and the collected material was frozen immediately. A voucher sample was deposited at the National Museum of Marine Biology and Aquarium, Taiwan (specimen No. 2011-03-1). The sponge (800 g, wet wt.) was stored frozen and then freeze dried. The freeze-dried material (250 g) was minced and extracted five times with EtOAc (1 L) for 24 h each time at room temperature. The organic extract was evaporated to yield a residue (22.5 g), which was subjected to open column chromatography on silica gel eluted with gradient solution of *n*-hexane (H)–EtOAc (E), to give 11 fractions: Fr-1 (eluted by *n*-hexane), Fr-2 (eluted by H–E 100:1), Fr-3 (eluted by H–E 50:1), Fr-4 (eluted by H–E 20:1), Fr-5 (eluted by H–E 10:1), Fr-6 (eluted by H–E 5:1), Fr-7 (eluted by H–E 3:1), Fr-8 (eluted by H–E 2:1), Fr-9 (eluted by H–E 1:1), Fr-10 (eluted by H–E 1:2), and Fr-11 (eluted by EtOAc). Fraction 8 (200 mg), was subjected to normal phase HPLC, using *n*-hexane–EtOAc (3:1) as the eluent, to afford four subfractions (A1–A4). Subfraction A1 (60 mg) was separated by normal phase HPLC using *n*-hexane–EtOAc (2:1) to afford DT (30.5 mg). The structure and the absolute configuration of this compound was unambiguously proven by X-ray diffraction analysis [47]. In the current report we isolated also aeroplysinin-1 and the other isomer of DT, (1′*R*,5′*R*,6′*S*)-2-(3′,5′-dibromo-1′,6′-dihydroxy-4′-oxo-cyclohex-2′-enyl) acetonitrile which was isolated in minute quantity [48,49]. 

A suitable colorless crystal was grown by slow evaporation of the EtOAc solution. Diffraction intensity data were acquired with Bruker APEX DUO single-crystal X-ray diffractometer with graphite-monochromated Mo Kα radiation (λ = 0.71073 Å). Crystal data for this compound: C_8_H_7_Br_2_NO_3_ (formula weight 324.97), approximate crystal size, 0.2 × 0.15 × 0.15 mm^3^, monoclinic, space group, P2_1_ (# 4), T = 100(2) K, a = 7.7217(3) Å, α = 90°, b = 8.2480(3) Å, β = 90°, c = 15.0536(6) Å, γ = 90°, V = 958.74(6) Å^3^, Dc = 2.251 mg/m^3^, Z = 4, F(000) = 624, µ_(MoKα)_ = 8.433 mm^−1^. A total of 8639 reflections were collected in the range 2.63 < θ < 26.67, with 1993 independent reflections [R(int) = 0.0202], completeness to θ_max_ was 99.6%; semi-empirical from equivalents absorption correction applied; full-matrix least-squares refinement on F^2^, the number of data/restraints/parameters were 1993/0/135; goodness-of-fit on F^2^ = 1.060; final R indices [I > 2 sigma (I)], R_1_ = 0.0134, wR_2_ = 0.0286; R indices (all data), R_1_ = 0.0143, wR_2_ = 0.0287, largest difference peak and hole, 0.311 and −0.282e/Å^3^. 

### 4.3. MTT Antiproliferative Assay

Cells were seeded at 4 × 10^4^ per well in 96-well culture plates before treatment with different concentrations of the test compound. After treatment for 24, 48, or 72 h, the cytotoxicity of the test compound was determined using MTT cell proliferation assay (thiazolyl blue tetrazolium bromide, Sigma-M2128). Light absorbance values (OD = OD_570_ − OD_620_) were recorded at wavelengths of 570 and 620 nm using an ELISA reader for calculating IC_50_, *i.e.*, the cell concentration at which the light absorbance value of the experimental group is half that of the control group. These results were expressed as a percentage of the control ± SD established from *n* = 4 wells per one experiment from three separate experiments.

### 4.4. Annexin V/PI Apoptosis Assay

The externalization of phosphatidylserine (PS) and membrane integrity were quantified using an annexin V-FITC staining kit. In brief, 10^6^ cells were grown in 35 mm diameter plates and were labeled with annexin V-FITC (10 µg/mL) and PI (20 µg/mL) prior to harvesting. After labeling, all plates were washed with a binding buffer and harvested. Cells were resuspended in the binding buffer at a concentration of 2 × 10^5^ cells/mL before analysis by flow cytometer FACS-Calibur (Becton-Dickinson, San Jose, CA, USA) and CellQuest software. Approximately 10,000 cells were counted for each determination.

### 4.5. Determination of ROS Generation and MMP Disruption

These assays were performed as described previously [50]. MMP disruption and ROS generation were detected with JC-1 cationic dye (5 µg/mL) and the carboxy derivative of fluorescein (carboxy-H_2_DCFDA, 1 mM), respectively. In brief, the treated cells were labeled with a specific fluorescent dye for 30 min. After labeling, cells were washed with PBS and resuspended in PBS at a concentration of 1 × 10^6^ cells/mL before analysis by flow cytometry.

### 4.6. Assay of Topoisomerase II Inhibitors and Poisons

The assay was performed as described previously [18]. Standard relaxation reaction mixtures (20 µL) containing 50 mMTris–HCl (pH 8.0), 10 mM MgCl_2_, 200 mM potassium glutamate, 10 mM dithiothreitol, 50 µg/mL bovine serum albumin, 1 mM ATP, 0.3 µg of pHOT1 plasmid DNA, two units of human topoisomerase II (Topogen, Columbus, OH, USA) and the indicated concentrations of etoposide and 10AB were incubated at 37 °C for 30 min. Reactions were terminated by the addition of 2 µL of 10% SDS to facilitate trapping the enzyme in a cleavage complex, followed by the addition of 2.5 µL of proteinase K (50 µg/mL) to digest the bound protein (incubated 37 °C for 15 min) and finally by adding 0.1 volume of the sample loading dye. The DNA products were analyzed by electrophoresis through vertical 2% agarose gels at 2 V/cm in 0.5XTAE buffer. Gels were stained with ethidium bromide and photographed using an Eagle Eye II system (Stratagene, La Jolla, CA, USA).

### 4.7. Western Blotting Analysis

Cell lysates were prepared by treating the cells for 30 min in RIPAlysis buffer, 1% Nonidet P-40, 0.5% sodium deoxycholate, 0.1% sodium dodecyl sulphate (SDS), 1 mM sodium orthovanadate, 100 µg/mL phenylmethylsulfonyl fluoride and 30 µg/mL aprotinin) (all chemicals were obtained from Sigma). The lysates were centrifuged at 20,000× *g* for 30 min, and the protein concentration in the supernatant was determined using a BCA protein assay kit (Pierce, Rockford, IL, USA). Equal amounts of proteins were respectively separated by 7.5%, 10% or 12% of SDS-polyacrylamide gel electrophoresis and then were electrotransferred to a PVDF membrane. The membrane was blocked with a solution containing 5% non-fat dry milk TBST buffer (20 mMTris-HCl, pH 7.4, 150 mM NaCl and 0.1% Tween 20) for 1 h and washed with TBST buffer. The protein expressions were monitored by immunoblotting using specific antibodies. These proteins were detected by an enhanced chemiluminescence kit (Pierce).

### 4.8. Statistics

The results were expressed as mean ± standard deviation (SD). Comparison in each experiment was performed using an unpaired Student’s *t*-test and * *P* value of less than 0.05 was considered to be statistically significant.

## 5. Conclusions

The purpose of this work was to elucidate the mechanism underlying the cytotoxic activity of marine dibromotyrosine, (1′*R*,5′*S*,6′*S*)-2-(3′,5′-dibromo-1′,6′-dihydroxy-4′-oxocyclohex-2′-enyl) acetonitrile (DT), against K562 cells. DT induced ROS production which resulted in mitochondrial dysfunction and apoptosis in K562 cells. Our results suggest that this marine product may elevate the ROS-induced apoptosis in leukemia K562 cells via the blockage of IKK/NFκB/HIF/PTEN pathway and the activation of PI3K/Akt pathway. Taken together, despite the large footprint of bromotyrosine derivatives we believe that they are still under-researched and our findings will provide an impetus for future research revealing the mechanisms underlying the biological activity of these compounds. 

## Supplementary Files

Supplementary File 1 Supplementary Materials (PDF, 101 KB)
