# Supplementary material for: Towards the Small and the Beautiful: A Small Dibromotyrosine Derivative from Pseudoceratina sp. Sponge Exhibits Potent Apoptotic Effect through Targeting IKK/NFκB Signaling Pathway
Source: Mar Drugs. 2013 Aug 26;11(9):3168–85. doi: 10.3390/md11093168 (PMC3801119; doi:10.3390/md11093168)
Supplement: Supplementary File 1 — Supplementary Materials (PDF, 101 KB) [file marinedrugs-11-03168-s001.pdf]

Supplementary Materials

Figure S1. Molecular plot of DT with confirmed absolute configuration.

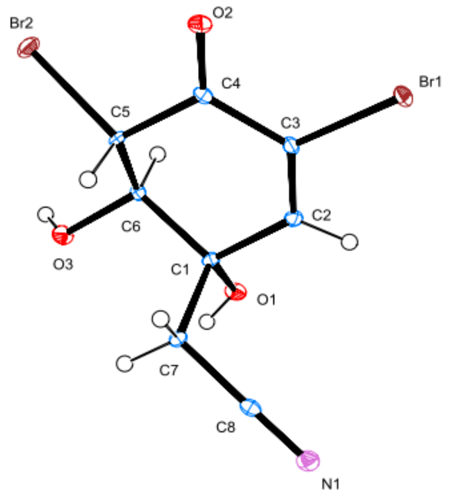

Table S1. Crystal data and structure refinement for sea21mo.

**Identification code**	**sea21mo**
Empirical formula	C_8H_7 Br_2NO_3
Formula weight	324.97
Temperature	100(2) K
Wavelength	0.71073 Å
Crystal system	Orthorhombic
Space group	P 21 21 21
	a = 7.7217(3) Å, α = 90 °
Unit cell dimensions	b = 8.2480(3) Å, β = 90 °
	c = 15.0536(6) Å, γ = 90 °
Volume	958.74(6) $Å^3$
Z	4
Density (calculated)	2.251 mg/m^3
Absorption coefficient	8.433 mm^{-1}
F(000)	624
Crystal size	0.20 × 0.15 × 0.15 mm^3
Theta range for data collection	2.63 to 26.67 °
Index ranges	$-9 \leq h \leq 9$, $-10 \leq k \leq 10$, $-18 \leq l \leq 18$
Reflections collected	8639
Independent reflections	1993 [R(int) = 0.0202]
Completeness to theta = 26.57 °	99.6%
Absorption correction	Semi-empirical from equivalents
Max. and min. transmission	0.9486 and 0.7803
Refinement method	Full-matrix least-squares on F^2
Data/restraints/parameters	1993/0/135
Goodness-of-fit on F^2	1.060
Final R indices [I > 2sigma(I)]	R1 = 0.0134, wR2 = 0.0286
R indices (all data)	R1 = 0.0143, wR2 = 0.0287
Absolute structure parameter	0.015(7)
Largest diff. peak and hole	0.311 and −0.282 e.$Å^{-3}$

Table S2. Atomic coordinates ($\times 10^4$) and equivalent isotropic displacement parameters ($Å^2 \times 10^3$) for sea21mo. U(eq) is defined as one third of the trace of the orthogonalized.

U^{ij} Tensor	*x*	*y*	*z*	U(eq)
Br(1)	3438(1)	2823(1)	4472(1)	17(1)
Br(2)	1810(1)	3577(1)	5436(1)	20(1)
O(1)	4634(2)	643(2)	7605(1)	14(1)
O(2)	1769(2)	447(2)	4370(1)	17(1)
O(3)	2509(2)	2208(2)	7374(1)	16(1)
N(1)	1932(2)	4026(2)	8057(1)	17(1)
C(1)	3240(3)	516(2)	6989(1)	10(1)
C(2)	3643(3)	1621(2)	6226(1)	13(1)
C(3)	3133(2)	1307(2)	5396(1)	12(1)
C(4)	2239(2)	200(2)	5125(1)	11(1)
C(5)	1861(3)	1367(2)	5881(1)	11(1)
C(6)	3105(3)	1248(2)	6667(1)	11(1)
C(7)	1560(2)	1078(2)	7456(1)	11(1)
C(8)	1760(3)	2737(2)	7798(1)	12(1)

Table S3. Bond lengths [Å] and angles [°] for sea21mo.

Bond	Bond length (Angle)
Br(1)–C(3)	1.8848(17)
Br(2)–C(5)	1.9423(18)
O(1)–C(1)	1.425(2)
O(1)–H(1)	0.72(2)
O(2)–C(4)	1.212(2)
O(3)–C(6)	1.405(2)
O(3)–H(3)	0.70(2)
N(1)–C(8)	1.141(2)
C(1)–C(2)	1.499(3)
C(1)–C(6)	1.538(3)
C(1)–C(7)	1.547(3)
C(2)–C(3)	1.335(3)
C(2)–H(2)	0.9300
C(3)–C(4)	1.480(3)
C(4)–C(5)	1.519(3)
C(5)–C(6)	1.526(3)
C(5)–H(5)	0.9800
C(6)–H(6)	0.9800
C(7)–C(8)	1.470(3)
C(7)–H(7A)	0.9700
C(7)–H(7B)	0.9700
C(1)–O(1)–H(1)	114(2)
C(6)–O(3)–H(3)	112(2)
O(1)–C(1)–C(2)	107.31(16)

Table S3. *Cont.*

O(1)–C(1)–C(6)	109.05(16)
C(2)–C(1)–C(6)	110.33(15)
O(1)–C(1)–C(7)	108.41(14)
C(2)–C(1)–C(7)	109.89(15)
C(6)–C(1)–C(7)	111.73(16)
C(3)–C(2)–C(1)	122.58(18)
C(3)–C(2)–H(2)	118.7
C(1)–C(2)–H(2)	118.7
C(2)–C(3)–C(4)	123.92(17)
C(2)–C(3)–Br(1)	121.68(14)
C(4)–C(3)–Br(1)	114.36(13)
O(2)–C(4)–C(3)	122.68(18)
O(2)–C(4)–C(5)	122.65(18)
C(3)–C(4)–C(5)	114.57(16)
C(4)–C(5)–C(6)	114.77(16)
C(4)–C(5)–Br(2)	109.87(12)
C(6)–C(5)–Br(2)	109.91(13)
C(4)–C(5)–H(5)	107.3
C(6)–C(5)–H(5)	107.3
Br(2)–C(5)–H(5)	107.3
O(3)–C(6)–C(5)	110.18(16)
O(3)–C(6)–C(1)	108.45(15)
C(5)–C(6)–C(1)	110.37(15)
O(3)–C(6)–H(6)	109.3
C(5)–C(6)–H(6)	109.3
C(1)–C(6)–H(6)	109.3
C(8)–C(7)–C(1)	110.49(16)
C(8)–C(7)–H(7A)	109.6
C(1)–C(7)–H(7A)	109.6
C(8)–C(7)–H(7B)	109.6
C(1)–C(7)–H(7B)	109.6
H(7A)–C(7)–H(7B)	108.1
N(1)–C(8)–C(7)	179.2(2)

Symmetry transformations used to generate equivalent atoms.

Table S4. Anisotropic displacement parameters ($Å^2 \times 10^3$) for sea21mo. The anisotropic displacement factor exponent takes the form: $-2\pi^2$ (h^2 a^{*2} U11 + ... + 2h k a* b* U12).

	U11	U22	U33	U23	U13	U12
Br(1)	23(1)	17(1)	11(1)	3(1)	2(1)	−6(1)
Br(2)	29(1)	11(1)	19(1)	−4(1)	−2(1)	−3(1)
O(1)	12(1)	20(1)	11(1)	−1(1)	−2(1)	0(1)
O(2)	21(1)	20(1)	10(1)	−2(1)	−1(1)	−3(1)
O(3)	23(1)	13(1)	13(1)	4(1)	2(1)	5(1)
N(1)	13(1)	18(1)	19(1)	−2(1)	1(1)	1(1)
C(1)	10(1)	11(1)	10(1)	−1(1)	−1(1)	1(1)
C(2)	11(1)	12(1)	15(1)	−3(1)	3(1)	0(1)
C(3)	13(1)	10(1)	13(1)	2(1)	5(1)	0(1)
C(4)	7(1)	14(1)	12(1)	0(1)	3(1)	2(1)
C(5)	15(1)	8(1)	11(1)	−4(1)	1(1)	−2(1)
C(6)	14(1)	10(1)	10(1)	1(1)	2(1)	−1(1)
C(7)	11(1)	11(1)	10(1)	−2(1)	−2(1)	−1(1)
C(8)	9(1)	18(1)	10(1)	1(1)	1(1)	2(1)

Table S5. Hydrogen coordinates ($\times 10^4$) and isotropic displacement parameters ($Å^2 \times 10^3$) for sea21mo.

	x	*y*	*z*	U(eq)
H(2)	4272	2561	6335	15
H(5)	699	−1116	6104	14
H(6)	4253	−1628	6483	14
H(7A)	604	1043	7038	13
H(7B)	1295	349	7943	13
H(1)	4430(30)	360(30)	8047(16)	23(8)
H(3)	2990(30)	−2930(30)	7412(15)	20(8)
